# Effect of Septimeb^TM^ as a new natural extract on severe sepsis: A randomized clinical trial

**Published:** 2017

**Authors:** Alieh Pourdast, Maryam Sanaei, Sirous Jafari, Mostafa Mohammadi, Hossein Khalili, Gita Shafiee, Zeinab Ahadi, Mahsa Rostami, Saba Alizad, Ramin Heshmat, Minoo Mohraz

**Affiliations:** 1 Department of Infectious and Tropical Diseases, Iranian Research Center for HIV/AIDS, Iranian Institute for Reduction of High-Risk Behaviors, Tehran University of Medical Sciences, Tehran, Iran.; 2 Chronic Diseases Research Center, Endocrinology and Metabolism Population Sciences Institute, Tehran University of Medical Sciences, Tehran, Iran.; 3 Department of Infectious and Tropical Diseases, Faculty of Medicine, Tehran University of Medical Sciences, Tehran, Iran.; 4 Department of Anesthesiology and Critical Care Medicine, , Imam Khomeini Hospital Complex, Faculty of Medicine, Tehran University of Medical Sciences, Tehran, Iran.; 5 Department of Pharmacotherapy , Imam Khomeini Hospital Complex, Faculty of Medicine, Tehran University of Medical Sciences, Tehran, Iran.; 6 Iranian Research Center for HIV/AIDS, Iranian Institute for Reduction of High-Risk Behaviors, Tehran University of Medical Science, Tehran, Iran.

**Keywords:** Sepsis, Septimeb, Infection, Anti-inflammatory effects

## Abstract

**Background:**

Septimeb as a herbal medicine has regulatory effects on inflammation. This study set to evaluate the effects of Septimeb among patients with sepsis on inflammatory biomarkers and survival rate.

**Methods:**

In this randomized clinical trial, 51 patients with sepsis from the ICU and medical ward of Imam Khomeini Hospital were divided into two groups: Septimeb (n=25) and control group (n=26). In the control group, the patients received a standard treatment only for 7 days, while Septimeb group received Septimeb (6cc vial with 500cc serum glucose infusion 5% daily for one to two hours) plus standard treatment of sepsis for 7 days. Then, blood samples were analyzed. APACHE (Acute Physiologic and Chronic Health Evaluation), SOFA (Sequential Organ Failure Assessment), and GCS (Glasgow Coma Score) values were calculated daily.

**Results:**

Treatment with Septimeb showed a significant decrease in SOFA value (1.54±0.83) compared to the control group (2.39±0.88) (P<0.001) and a significant increase in GCS value (14.46±0.88) compared to the control group (12.86±1.78) (P<0.001). Improvements of these values can confirm the potential of Septimeb in the reduction of severity of sepsis (P<0.05). There were significant decreases in lactate and blood sugar and WBC levels. In addition, inflammatory factors such as ESR (Septimeb group: 52.07±34.80, control group: 51.75±42.10, P=0.98) and CRP (Septimeb group: 48.86±23.21, control group: 49.93±36.22, P=0.92) decreased, but did not show a significant reduction.

**Conclusion:**

Septimeb has positive effects on reduction of the severity of sepsis which leads to reduction of patients’ mortality rates.

Sepsis is a bacterial infection in the circulation and body tissues (1). Sepsis results from numerous types of microscopic disease-causing organisms. This is an increasingly common cause of morbidity and mortality (2).This infection influences over 18 million people worldwide and is expected to increase by 1% per annum (3, 4). It is one of the most prevalent and deadly diseases worldwide even in the most developed countries (2, 5). Clinical manifestations of bacterial sepsis are: body temperature abnormalities, irregularities of heart and respiratory rate. There have been two clinical syndromes related to sepsis such as septic shock and severe sepsis. Septic shock is an extremely deadly syndrome of cardiovascular shock, resulting in death in only about 24-48 hours since onset. Severe sepsis is associated with the dysfunction of organs such as hypoxemia, oliguria, lactic acidosis, and liver enzymes (6, 7). Severe sepsis is an important complication of infection that seeks to stimulate the inflammatory and coagulation system.

Studies have shown that proinflammatory cytokines such as tumor necrosis factor (TNF) or high-mobility group box1 (HMGB1) are powerfully released and can cause organ and tissue dysfunction. Therefore, it seems that inhibition of proinflammatory cytokines could likely prevent inflammatory responses and organ impairment (8, 9). 

Septimeb is a herbal medicine and an inflammation regulator. Septimeb is a combination of the extracts from Rosa canina, Urtica dioica (nettle), Tanacetum vulgare (tansy), selenium, flavonoids, and carotenes (10). It reduces inflammatory cytokines, TNF-alpha, existence of selenium in the core, and the positive effects on the induction of life at the cellular level (11). A study has shown a safe dose of IMODTM (a weaker form of Septimeb) that can be used in future clinical trials (12). Mahmoodpoor et al. showed that IMOD was significantly effective in improving SOFA and APACHE values and decrease of mortality rate (13). The objective of this study was to evaluate the effect of Septimeb on plasma levels of inflammatory biomarkers and mortality rate in patients with sepsis.

## Methods

**Figure 1:**
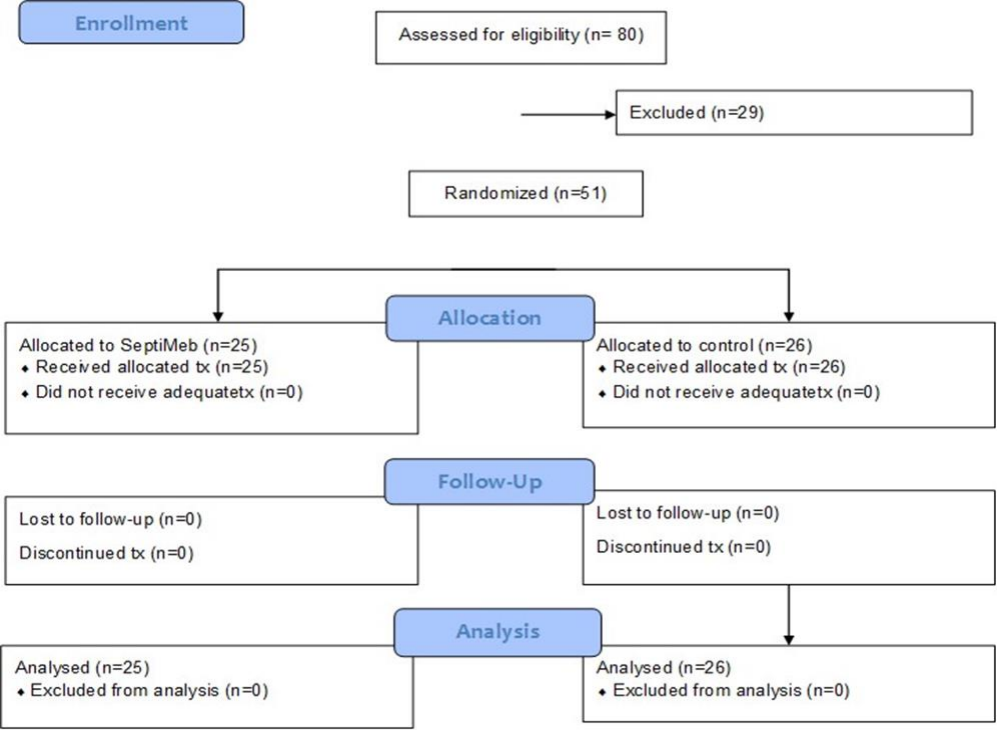
CONSORT trial flow diagram for patients with sepsis who were accrued into the trial.

**Study design and subjects:** This was a clinical trial study carried out on 51 patients with sepsis to assess the effects of Septimeb on inflammatory biomarkers and survival rate. The study protocol was approved by the Ethics Committee of Tehran University of Medical Sciences (TUMS). In this study, patients with sepsis were enrolled from September 2013 to December 2014 and admitted to medical ward and general ICU of Imam Khomeini Hospital of TUMS. Exclusion criteria were age below 18 and above 65 years old, hypersensitivity, pregnancy or breast feeding, kidney, liver and heart failure, cancer, immune system deficiency, vascular problems, and specific infections. Permuted balanced block randomization was used as the research method. Fifty one patients with sepsis were divided into two groups of Septimeb (n=25) and control (n=26) (figure 1). In the control group, patients received standard treatment and in the intervention group, subjects received Septimeb (A 6cc vial with 500cc serum glucose infusion 5% daily for one to two hours) plus standard treatment of sepsis for 7 days. 

**Measurement:** Blood factors (white blood cell: WBC, Hematocrit, and platelets) were assessed by hematology analyzer or cell counter. Biochemical analyzer was used for biochemical tests (blood sugar, Na, K, calcium, urea, creatinine, and lactate) through venous blood sampling. Blood samples were collected into tubes containing EDTA and without EDTA. The samples without EDTA were spun at 3000 × g for 15 minutes to separate serum from blood. Then patients’ plasma was stored at −80°C until the time of analysis. CBC samples containing EDTA were measured by hematology analyzer. Blood gases (PH, PO2, PCO2, CO2, and HCO3) were assessed by blood gas analyzer (arterial blood). APACHE (Acute Physiology and Chronic Health Evaluation), GCS (Glasgow Coma Scale), and SOFA (Sequential Organ Failure Assessment) values were measured. SOFA is used to calculate the value and intensity of organ impairment in six organ systems (coagulation, respiratory liver, cardiovascular, neurologic and renal). An increase in SOFA during the first 24-48 hours in the ICU forecasts a mortality rate of at least 50% to 95% (14-17). APACHE is a severity-of-illness classification system; higher values of which correspond to more severe disease and a higher risk of death (18, 19). GCS is used to assess the status of CNS (central nervous system). The minimum possible GCS score is 3 (deep coma or death), while the maximum is 15 (fully awake person) (20, 21). In our study, primary outcomes included APACHE, SOFA, and GCS, and secondary outcomes included vital signs, hematologic, inflammatory markers, biochemical variables, and arterial blood gas (ABG). 

**Patient ethical considerations:** Initially, a consent form was obtained from all patients or their legal guardian. Our clinical trial was registered in IRCT (Iranian Registration Clinical Trial) with registration number: of (IRCT201108014076N3). 

Mean (standard deviation: SD) values for continuous and frequencies (%) for categorical variables of the baseline characteristics variables were compared between two groups using student's *t*-test and *x2* test, respectively. The differences of survival rates among the two groups were analyzed by Kaplan- Meier (Log rank) test. P-values less than 0.05 were considered statistically significant. All data analyses were carried out using SPSS 18 PASW Version.

## Results

The comparison of differences in baseline values of vital signs, hematologic, inflammatory markers and biochemical and arterial blood gas for the control and Septimeb groups has been summarized in table 1. There was not a significant difference between two groups in baseline values.

**Table 1:** Comparison of vital signs, hematologic and biochemical parameters between groups in baseline

**Variables**	**Control group**	**Septimeb group**	***P*** **- value**
**Vital Signs**			
Body Temperature (^◦^C) Heart Rate (beats/min) Respiratory Rate (breaths/min) Arterial pressure (mmHg) Systolic Blood pressure (mmHg) Diastolic Blood pressure (mmHg)	38.59±0.44 107.57±15.89 23.32±6.60 88.46±18.96 119.93±22.34 74.50±13.17	38.69±0.50 108.36±15.95 24.24±7.86 90.12±18.73 116.08±15.01 73.87±9.98	0.45 0.60 0.98 0.77 0.48 0.85
**Hematologic, ** **inflammatory ** **and biochemical markers**			
WBC (× 10^3^) cells/mm^3^ Hematocrit Platelets (× 10^3^) microL^–1^ Sedimentation CRP PT PTT Blood Sugar (mg/dL ) Sodium (meq/l) Potassium (meq/l) Calcium (mg/dL ) Urea (mg/dL ) Creatinine (mg/dL ) Lactate (mg/dL)	15.83±3.59 31.46±7.30 275.72±109.24 65.52±36.33 62.17±28.23 21.46±28.59 29.63±7.60 163.77±65.62 137.36±5.29 4.17±0.52 8.25±0.93 33.32±25.04 0.86±0.45 606.35±223.91	18.15±6.00 33.08±6.28 162.89±125.81 68.00±32.04 56.90±22.09 22.25±30.50 29.28±9.94 187.00±37.61 137.30±5.76 4.31±0.65 8.46±0.65 36.96±30.22 0.99±0.38 627.35±249.24	0.12 0.57 0.001 0.82 0.51 0.93 0.67 0.28 0.97 0.39 0.86 0.64 0.28 0.78
**Arterial blood gas**			
PH HCO3 (meq/l) Pa O_2 _(mmHg) Pa CO_2 _(mmHg)	7.40±0.13 23.09±4.59 50.38±27.5 34.55±13.52	7.40±0.14 21.90±6.09 49.70±19.64 34.44±12.88	0.97 0.43 0.92 0.98

WBC: white blood cell, CRP: C - reactive protein test, Data are mean ± Standard Deviation (SD); The significance level P<0.05

Table 2 shows the comparison of baseline and day 7 of vital signs, hematologic and inflammatory markers, and biochemical and arterial blood gas in each group, separately. There was a significant decrease in body temperature on the seventh day of the control group (37.62±1.04) compared to baseline (38.59±0.44). In addition, a significant decrease in body temperature on the seventh day of Septimeb group (36.71±0.44) was observed compared to baseline (38.69±0.50) (p<0.05). 

There were significant reductions in heart rate and as well respiratory rate on the seventh day compared to the first day in both groups (table 2). There was a substantial decrease in the level of platelets on the seventh day in the control group (187.68±135.32) compared to the first day (275.72±109.24); a significant increase was observed in the level of platelets in the seventh day of Septimeb group (287.60±224.52) compared to the first day (162.89±125.81) (p<0.05). 

**Table 2:** Comparison for Vital signs, hematologic, biochemical and a between first day and 7th day of study in two groups

Variables	**Control group**		**P-value**	**Septimeb group**		P-value
	**Baseline**	**7th day**		**Baseline**	**7th day**	
**Vital Signs**						
Body Temperature (^◦^C) Heart Rate(beats/min) Respiratory Rate (beats/min) Arterial pressure (mmHg) Systolic Blood pressure (mmHg) Diastolic Blood pressure (mmHg)	38.59±0.44 107.57±15.89 23.32±6.60 88.46±18.96 119.93±22.34 74.50±13.17	37.62±1.04 93.39±15.95 19.39±4.90 86.38±20.65 117.68±14.52 73.43±10.13	<0.001 <0.001 0.02 0.62 0.62 0.70	38.69±0.50 108.36±15.95 24.24±7.86 90.12±18.73 116.08±15.01 73.87±9.98	36.71± 0.44 90.29±11.78 18.46±4.79 85.25±11.36 114.58±12.74 72.00±7.67	<0.001 <0.001 0.004 0.39 0.49 0.42
**Hematologic, ** **inflammatory ** **and biochemical markers**						
WBC(×10^3^) cells/mm^3^ Hematocrit Platelets (×10^3^) microL^–1^ Sedimentation CRP PT PTT Blood Sugar (mg/dL) Sodium (meq/l) Potassium (meq/l) Calcium(mg/dL) Urea (mg/dL) Creatinine (mg/dL) Lactate (mg/dL)	15.83±3.59 31.46±7.30 275.72±109.24 65.52±36.33 62.17±28.23 21.46±28.59 29.63±7.60 163.77±65.62 137.36±5.29 4.17±0.52 8.25±0.93 33.32±25.04 0.86±0.45 606.35±223.91	11.78±5.04 30.10±6.09 187.68±135.32 51.75±42.10 49.93±36.22 28.05±54.47 32.19±18.61 132.73±47.75 133.94±22.68 4.14±0.68 7.86±1.73 34.32±21.71 1.58±4.02 539.62±269.37	<0.001 0.07 0.008 0.006 0.05 0.61 0.47 0.01 0.45 0.78 0.25 0.82 0.32 0.79	18.15±6.00 33.08±6.28 162.89±125.81 68.00±32.04 56.90±22.09 22.25±30.50 29.28±9.94 187.00±37.61 137.30±5.76 4.31±0.65 8.46±0.65 36.96±30.22 0.99±0.38 627.35±249.24	10.15±4.70 31.18±6.18 287.60±224.52 52.07±34.80 48.86±23.21 12.84±2.60 36.54±28.86 121.90±44.67 136.33±5.61 7.02±0.76 7.99±1.98 26.37±18.36 1.16±1.49 376.95±194.57	<0.001 0.06 0.04 0.06 0.10 0.16 0.13 0.03 0.52 0.19 0.33 0.03 0.56 0.04
**Arterial blood gas**						
PH HCO3 (meq/l) Pa O_2 _(mmHg) Pa CO_2 _(mmHg)	7.40±0.13 23.09±4.59 50.38±27.5 34.55±13.52	7.17±0.21 22.55±5.67 53.94±30.31 32.30±10.91	<0.001 0.56 0.68 0.47	7.40±0.14 21.90±6.09 49.70±19.64 34.44±12.88	7.20±0.21 21.19±4.45 46.27±20.51 34.30±10.50	0.001 0.68 0.30 0.51

Data are mean ± Standard Deviation (SD); the significance level P<0.05

A decrease was observed in the levels of blood sugar in both groups on the seventh day though the decrease in control group was rather more than the Septimeb group (p<0.05). Even more, a substantial decrease was observed in urea levels in Septimeb group. There was an increase in urea levels in the control group which was not statistically significant. A significant decrease was observed in lactate levels in Septimeb group on the seventh day. But there was an increase of lactate levels which was not statistically significant in the control group at the end of the intervention. A significant decrease was observed in PH in both groups at the end of intervention (seventh day) (table 2). Table 3 shows the comparison of vital signs, hematologic, biochemical, and arterial blood gas between the control group and Septimeb group on the seventh day. The body temperature attributed to Septimeb group on the seventh day (36.71**±**0.44) was significantly less than the control group (37.62**±**1.04). 

Lactate levels in Septimeb groups (376.95±194.57) were significantly less than lactate levels of the control group (539.62±269.37) on the seventh day. Table 4 shows changes in APACHE, SOFA, and GCS values from the first day to the seventh day of study. The mean difference of SOFA in Septimeb group (1.54±0.83) was significantly reduced compared to the control group (2.39±0.88). There was a significant increase in GCS of patients of Septimeb group (14.46 ±0.88) compared to the control group (12.86±1.78) (p<0.05) from the first day to the seventh day.

**Table 3:** Comparison of vital signs, hematologic, biochemical and arterial blood gas of two groups on 7th day

**Variables**	**Control group 7th day**	**Septimeb group 7th day**	**p-value**
**Vital Signs**			
Body Temperature (^◦^C) Heart Rate (beats/min) Respiratory Rate (beats/min) Arterial pressure (mmHg) Systolic Blood pressure (mmHg) Diastolic Blood pressure (mmHg)	37.62±1.04 93.39±15.95 19.39±4.90 86.21±19.85 117.68±14.52 73.43±10.13	36.71± 0.44 90.29±11.78 18.46±4.79 85.25±11.36 114.58±12.74 72.00±7.67	<0.001 0.43 0.49 0.90 0.42 0.57
**Hematologic and Biochemical**			
WBC(× 10^3^) cells/mm^3^ Haematocrit Platelets (× 10^3^) microL^–1^ Sedimentation CRP Blood Sugar (mg/dL) Sodium (meq/l) Potassium (meq/l) Calcium (mg/dL) Urea (mg/dL) Creatinine (mg/dL) Lactate (mg/dL)	11.78±5.04 30.10±6.09 187.68±135.32 51.75±42.10 49.93±36.22 132.73±47.75 133.94.±22.68 4.14±0.68 7.86±1.73 34.32±21.71 1.58±4.02 539.62±269.37	10.15±4.70 31.18±6.18 287.60±224.52 52.07±34.80 48.86±23.21 121.90±44.67 136.33±5.61 7.02±0.76 7.99±1.98 26.37±18.36 1.16±1.49 376.95±194.57	0.24 0.54 0.08 0.98 0.92 0.13 0.62 0.12 0.99 0.16 0.64 0.05
**Arterial blood gas**			
PH HCO3(meq/l) Pa O_2 _(mmHg) Pa CO_2 _(mmHg)	7.17±0.21 22.55±5.67 53.94±30.31 32.30±10.91	7.20±0.21 21.19±4.65 46.27±20.51 34.30±10.50	0.62 0.36 0.31 0.51

WBC: white blood cell; CRP:C - reactive protein Test, Data are mean ± Standard Deviation(SD)

The significance level P<0.05

**Table 4:** Change in APACHE, SOFA and GCS from first day to 7th day study

	**Control group**	**Septimeb group**	**P-value**
APACHE	7.58±4.37	6.79±3.69	<0.36
SOFA	2.39±0.88	1.54±0.83	0.001
GCS	12.86±1.78	14.46±0.88	<0.001

APACHE: Acute Physiologic and Chronic Health Evaluation, SOFA: Sequential Organ Failure Assessment, GCS: Glasgow Coma Score, Data are mean differences between 7^th^ and first day ± Standard Deviation (SD)

The significance level P<0.05

**Figure 2:**
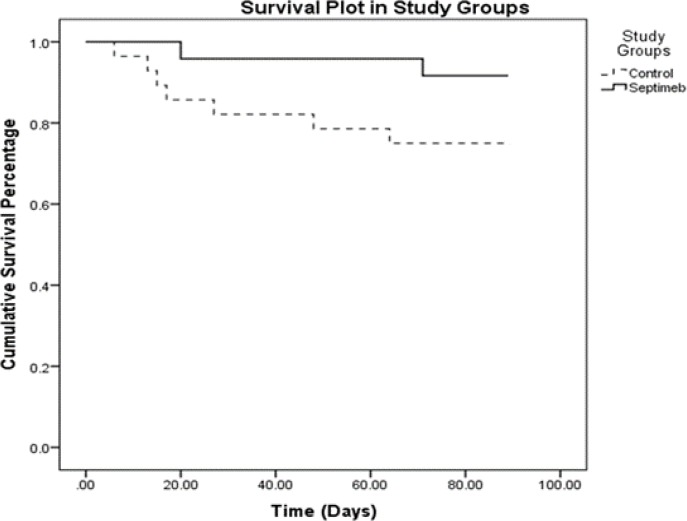
Changes in survival of patients in the control and Septimeb groups

Figure 2 displays the liberation of patients’ survival at 3-month follow-up. Death in the control group was 7 (25%) and in Septimeb was 2 (8.3%).

## Discussion

This study showed that the Septimeb with standard treatment of severe sepsis could increase survival ratio and reduces mortality rate in patients. The comparison of survival time indicated that the number of deaths in the Septimeb group was lower than the control group. Survival rate refers to percentage of people in a study or treatment group still alive for a given period of time after diagnosis. Therefore, this result may be very valuable in the clinical settings.

The outcome revealed that the SOFA and GCS values of Septimeb group significantly improved at the end of the intervention compared to the control group. It was found that the APACHE improved (but not significantly). The findings of other studies proved to be consistent with the current study, showing that Septimeb can reduce APACHE and SOFA(12, 13). Lactate and urea levels in Septimeb group decreased considerably compared to the control group. There was a significant decrease of lactate level in Septimeb group compared to the control group on the seventh day. Urea, blood sugar, and CRP levels of patients in Septimeb group decreased, but not significantly.

Septimeb is a herbal drug and a stronger form of IMOD^TM^. It is a combination of the extracts of Rosa canina, Urtica dioica (nettle), Tanacetum vulgare (tansy), selenium, flavonoids, and carotenes (10, 22). Rosa canina has effective role on the blood sugar and cholesterol level regulation. Tanacetum vulgare has anti-inflammatory effects (10). Urtica dioica extract is likely to prevent maturation of myeloid dendritic cells and reduce T cell responses (10). Experimental studies have shown that Septimeb can be regulator of TNF-α, interferon-γ (IFN-γ), and IL-2 (11). In vivo and in vitro studies have revealed that IMOD^TM ^can influence on immunogenic diabetes type 1 and inflammatory bowel diseases (10, 12, 13, 23-27). 

Other studies in 2007 and 2010 showed that IMOD is harmless and has no allergenic and mutagenic potentials (12, 13). Potential effects of IMOD™ have been shown in HIV patients on decreasing TNF-α and IL-2 levels (28). There is continuous increase of multiple cytokine in severe sepsis. They are important inflammatory mediators (29-32), leading to uncontrolled inflammation. Removing the inflammatory cascade can improve survival. Besides are large number of cytokines including TNF-α, IL-1, and IL-6 (28, 33). Cytokines are polypeptide hormones. At the molecular level, production of CRP is induced by proinflammatory cytokines in the liver (34). Cytokines are produced by steroid hormones, neuropeptides, and bacterial components (35). In this study, CRP level was very low in Septimeb group. It appeared that the Septimeb drug reduced the inflammatory factors, but it was not significant due to the small number of patients and short duration of follow-up.

Imani et al. showed that WBC, ESR, and CRP rise considerably during sepsis. These factors decreased after recovery (36). Based on the findings, the level of WBC decreased significantly at the end of the intervention compared to the first day in Septimeb group; however, this decline was seen in the control group as well. More reduction was observed in Septimeb group compared to the control group. The ESR and CRP levels were low compared to the control group, suggesting that inflammation is reduced. These factors decreased, but not to a great extent, likely, because of the small number of patients and short-term follow-up. Improvement of these values can confirm the potential of Septimeb in reduction of the severity of sepsis and consequently in the reduction of patients’ mortality rates (14). Moreover, previous studies have not shown improvement in patients with sepsis (13). Eslami et al. showed that Septimeb has positive effects on mortality rate in severe sepsis (22) and indicated that improving tissue oxygenation could be a reason of Septimeb in the decrease of mortality rate. 

Douzinas et al showed that production of lactate and cytokines in sepsis are related to organ failure (37). Another study has shown that lactate serum is related to mortality rate in severe sepsis, independent of organ failure and shock (38). Lactate is an unreliable index of tissue hypoxia in sepsis(39). In this study, lactate level was significantly lower at the end of intervention in Septimeb group compared to the control group. Therefore, improving tissue oxygenation could be a probable mechanism in reduction of sepsis mortality after drug intervention with Septimeb. The current study suffered some limitations: The number of participants taking part in the study was low; Potential random error could not be removed completely. Study strengths: Septimeb is produced inside the country, and it is a herbal drug. Consequently, Septimeb may have a considerable impact in reducing hospital costs. The unstable and anaphylactic hemodynamic responses were not observed during intervention.

In conclusion, Septimeb had positive effect on lactate, SOFA, and GCS values which are the indices of recovery in patients with sepsis, according to the significant impact of this herbal drug on the improvement of the disease, hence it can recommend using Septimeb with standard treatment. Septimeb may have a considerable effect in reducing mortality rate and better results can be obtained if the follow-up duration of this study was longer.
